# Hypocholesterolemic Properties and Prebiotic Effects of Mexican *Ganoderma lucidum* in C57BL/6 Mice

**DOI:** 10.1371/journal.pone.0159631

**Published:** 2016-07-20

**Authors:** María E. Meneses, Daniel Martínez-Carrera, Nimbe Torres, Mónica Sánchez-Tapia, Miriam Aguilar-López, Porfirio Morales, Mercedes Sobal, Teodoro Bernabé, Helios Escudero, Omar Granados-Portillo, Armando R. Tovar

**Affiliations:** 1 CONACYT–Colegio de Postgraduados, *Campus* Puebla, Puebla, Puebla, México; 2 Biotecnología de Hongos Comestibles, Funcionales y Medicinales, Colegio de Postgraduados (CP), *Campus* Puebla, Puebla, Puebla, México; 3 Departamento de Fisiología de la Nutrición, Instituto Nacional de Ciencias Médicas y Nutrición Salvador Zubirán (INCMNSZ), Ciudad de México, México; University of Catania, ITALY

## Abstract

Edible and medicinal mushrooms contain bioactive compounds with promising effects on several cardiovascular risk biomarkers. However, strains of *Ganoderma lucidum* of Mexican origin have not yet been studied. Standardized extracts of *G*. *lucidum* (*Gl*) were given to C57BL/6 mice fed a high-cholesterol diet compared with the drug simvastatin. The effects of the extracts on serum biochemical parameters, liver lipid content, cholesterol metabolism, and the composition of gut microbiota were assessed. Acetylsalicylic acid (10 mM) added to the cultivation substrate modulated properties of *Gl* extracts obtained from mature basidiomata. Compared to the high-cholesterol diet group, the consumption of *Gl* extracts significantly reduced total serum cholesterol (by 19.2% to 27.1%), LDL-C (by 4.5% to 35.1%), triglyceride concentration (by 16.3% to 46.6%), hepatic cholesterol (by 28.7% to 52%) and hepatic triglycerides (by 43.8% to 56.6%). These effects were associated with a significant reduction in the expression of lipogenic genes (Hmgcr, Srebp1c, Fasn, and Acaca) and genes involved in reverse cholesterol transport (Abcg5 and Abcg8), as well as an increase in Ldlr gene expression in the liver. No significant changes were observed in the gene expression of Srebp2, Abca1 or Cyp7a1. In several cases, *Gl*-1 or *Gl*-2 extracts showed better effects on lipid metabolism than the drug simvastatin. A proposed mechanism of action for the reduction in cholesterol levels is mediated by α-glucans and β-glucans from *Gl*, which promoted decreased absorption of cholesterol in the gut, as well as greater excretion of fecal bile acids and cholesterol. The prebiotic effects of *Gl*-1 and *Gl-*2 extracts modulated the composition of gut microbiota and produced an increase in the *Lactobacillaceae* family and *Lactobacillus* genus level compared to the control group, high-cholesterol diet group and group supplemented with simvastatin. Mexican genetic resources of *Gl* represent a new source of bioactive compounds showing hypocholesterolemic properties and prebiotic effects.

## Introduction

The prevalence of overweight and obesity has increased considerably in countries with a food pattern transition based on the consumption of processed foods. Unfortunately, this transition leads to numerous health problems and obesity-related disorders, such as the development of cardiovascular diseases (CVD), one of the major causes of death worldwide [1]. Epidemiological studies have shown that elevated total plasma cholesterol levels cause hyperlipidemia and increased risk of CVD [2]. In the third report of the National Cholesterol Education Program (NCEP), the Adult Treatment Panel III (ATP III) guidelines proposed to reduce plasma cholesterol concentrations, including lower intakes of dietary cholesterol (<200 mg/day) and saturated fat (<7% of calories), as well as an increase in viscous soluble fiber (10–25 g/day) [3]. Recent studies have demonstrated that dietary strategies play a central role in the prevention of atherosclerosis, focusing on functional foods containing bioactive compounds capable of adjusting lipid profiles to healthy levels through cholesterol and lipoprotein metabolism [4].

Cholesterol homeostasis in the body is mainly controlled by the endogenous synthesis, intestinal absorption, and hepatic excretion of dietary cholesterol. These processes are mediated by several transcription factors, which are fundamental to understanding the regulation of cholesterol metabolism. Sterol regulatory element-binding protein 2 (Srebp2) is the key regulator of gene expression linked to cholesterol synthesis, including the rate-limiting enzyme 3-hydroxy-3-methylglutaryl coenzyme A reductase (Hmgcr). Srebp2 activation increases the expression of the low-density lipoprotein receptor (Ldlr), which regulates the liver tissue uptake of LDL-C. Furthermore, hepatic cholesterol in the form of oxysterols represents a group of ligands for the liver X receptor (LXR) transcription factor, which upregulates the expression of the sterol regulatory element binding transcription factor 1c (Srebp1c) [5]. This factor activates the expression of lipogenic genes [6]. Isoforms of LXR, LXR-α and LXR-β, are capable of increasing the expression of several genes involved in bile acid synthesis, such as cholesterol 7 alpha-hydroxylase (Cyp7a1), as well as those associated with reverse cholesterol transport, including the ATP-binding cassette subfamily G members 5 and 8 (Abcg5 and Abcg8) and the ATP-binding cassette subfamily A (Abca1), among others.

Recent evidence suggests that high serum cholesterol concentration can be treated using a different factor not previously associated with cholesterol metabolism, the gut microbiota [7]. Further analyses have shown the important role of microbiota in regulating whole-body cholesterol homeostasis and its direct association with the development of atherosclerotic CVD [8, 9]. During dysbiosis, there is an increase in the permeability of the gut barrier, allowing the entrance of lipopolysaccharides and activating toll-like-4 receptors (TLR-4). These processes lead to the inhibition of LXR activation, resulting in an imbalance in cholesterol metabolism [10].

Edible, functional, and medicinal mushrooms contain nutrients and bioactive compounds with well-documented effects on several cardiovascular risk biomarkers [11]. The hypocholesterolemic effects of mushrooms relevant to CVD involve lipid and lipoprotein metabolism, anti-inflammation, and the inhibition of oxidative damage and platelet agglutination. In the case of the medicinal mushroom *Ganoderma lucidum*, known commercially as “Lingzhi” or “Reishi”, more than 300 triterpenes, polysaccharides (*e*.*g*., β-(1→3)-D-glucan), ganoderic acids, proteins, peptides, steroids, sterols and other bioactive compounds have been shown to result in pharmacological activities. Some of these compounds show promising hypolipidemic, hepatoprotective, antioxidative, antiatherosclerotic, and antiinflammatory effects [12–14]. However, comparative analyses of most research studies are difficult because species identification is unclear in numerous cases, and the chemical composition of *Ganoderma*-based products/compounds used is heterogeneous or shows diverse levels of purification and elaboration. Furthermore, virtually all scientific studies reporting medicinal properties of *G*. *lucidum* have used strains from Southeast Asia. For these reasons, the authors began a bioprospection research program to investigate novel functional and medicinal properties of edible mushrooms based on Mexican genetic resources, considering the great and unique biodiversity of this region. Native strains of *G*. *lucidum* have been isolated and identified at the molecular level. Hydroalcoholic extracts have been characterized in terms of nutrient composition and antimicrobial properties, and a strategy has been developed to modulate their functional properties through acetylsalicylic acid [15].

Thus, the aim of the present study was to analyze the effects of two hydroalcoholic extracts obtained from basidiomata of a Mexican strain of *G*. *lucidum*, which had never been studied, on total plasma cholesterol levels. Low and high doses of these standardized extracts were given to C57BL/6 mice fed a high-cholesterol diet according to a dietary approach compared with the drug simvastatin. Furthermore, to establish the possible molecular mechanisms responsible for these changes, their effects on lipid accumulation in the liver, gene expression in the liver (lipogenesis, endogenous cholesterol synthesis, reverse cholesterol transport, and bile acid synthesis), the excretion of fecal bile acids and cholesterol, and the composition of gut microbiota were assessed. Several bioactive compounds were associated with a proposed mechanism of action showing hypocholesterolemic properties and prebiotic effects of extracts of *G*. *lucidum* in an *in vivo* model.

## Materials and Methods

### Cultivation of *Ganoderma lucidum* (*Gl*), standard extracts, and chemical characteristics

#### Mushroom cultivation and harvesting

The CP-145 strain of *Ganoderma lucidum* (Curtis) P. Karst. was isolated by tissue culture from a wild basidioma growing on dead tree in the State of Morelos, Mexico (2,300 m altitude). This strain was deposited at the Centre for Genetic Resources of Edible, Functional and Medicinal Mushrooms (CREGEN-HCFM), CP, *Campus* Puebla, Mexico. The gene sequence of the ITS1-5.8S-ITS2 region from the rDNA of strain CP-145 was deposited at GenBank (www.ncbi.nlm.nih.gov/genbank/), under accession number LN998989. The strain was maintained and subcultured on malt extract agar medium (MEA, Bioxon), routinely autoclaved at 121°C for 15 min, and incubated at 28–29°C. Wheat grain spawn was prepared according to standard methods. Oak (*Quercus acutifolia* Née) sawdust was introduced into polypropylene plastic bags (47 x 13.5 cm) with a microfilter of 0.5 microns to allow gas exchange (Unicorn Imp. & Mfg. Corp., U.S.A.). Two groups of bags were prepared for mushroom cultivation, control and treatment, according to previously described methods [15]. Distilled water (1,300 ml) was added to each control bag containing 1,000 g of dry oak sawdust and was mixed homogeneously. A solution of acetylsalicylic acid (ASA, 10 mM; Sigma-Aldrich, U.S.A.) in distilled water (1,300 ml) was added to every treated bag containing 1,000 g of dry oak sawdust and was mixed homogeneously. All bags (2 kg wet substrate per bag) were sterilized at 121°C for 90 min. After cooling under aseptic conditions, sterilized substrates were inoculated by uniform mixing at a rate of *ca*. 50 g of CP-145 spawn per kg of fresh substrate weight (5%, w/w). The inoculated plastic bags were sealed and placed on shelves for incubation in the dark (24°-25°C). The bags were moved to the fruiting room when fully colonized by the mushroom mycelium and were maintained under constant conditions of temperature (20°-25°C), relative humidity (60–70%), and natural ventilation. The roof had two rows of transparent plastic sheets, which allowed indirect daylight (approximately 12 h) during the fruiting cycle. Mature basidiomata from each bag were harvested, cut into slices (*ca*. 1–2 cm), dried at 40°C in a forced air drying oven (SMO28-2, Shel Lab, U.S.A.) for five days, and stored at -80°C inside plastic bags.

#### Standardized mushroom extracts

Dried mushroom slices were chopped in a blender, and 10 g of the product was placed in a filter paper (8 μm) bag for maceration (24 h). Hydroalcoholic extracts (32% by volume) were obtained according to a previous patent (MX322035-B, July 8, 2014, Martínez-Carrera et al.). Mushrooms extracts were concentrated to 10 ml in a rotary evaporator (HS-2000NS, Hahn Shin Scientific, South Korea) at 19°C. They were then filter-sterilized (0.45 μm, Merck Millipore, Mexico), freeze-dried overnight using a vacuum apparatus (Labconco, Freezone 4.5, U.S.A.), and stored at 4°C until use. In this study, standardized extracts of *G*. *lucidum* cultivated on the control substrate were labeled *Gl*-1, whereas those cultivated on the treated substrate (ASA, 10 mM) were labeled *Gl*-2.

#### Nutrient composition of *Gl* extracts

The nutrient composition of extracts of *G*. *lucidum* (*Gl*-1 and *Gl*-2) was analyzed according to standard protocols and is shown in S1 Table and S1 Fig.

### Animals and treatments

Fifty-six male C57BL/6 mice, seven weeks old, weighing 26 g ± 0.50, were obtained from the animal facility at the Instituto Nacional de Ciencias Médicas y Nutrición Salvador Zubirán (INCMNSZ). The mice were housed at 23 ± 2°C, relative humidity of 45–55%, on a 12-hour light/12-hour dark cycle. Mice were randomly assigned to one of the following seven experimental groups (n = 8): 1) Ctrl: Control diet (AIN-93) [16]; 2) Ch: High-cholesterol diet (0.5% cholesterol) (Sigma-Aldrich, New Zealand); 3) Ch+Sim: High-cholesterol diet (0.5%) + simvastatin (0.03 g/100 g); 4) Ch+*Gl*-LD-1: High-cholesterol diet (0.5%) + *Gl*-1 low dose (0.5%); 5) Ch+*Gl*-HD-1: High-cholesterol diet (0.5%) + *Gl-*1 high dose (1.0%); 6) Ch+*Gl*-LD-2: High-cholesterol diet (0.5%) + *Gl-*2 low dose (0.5%); and 7) Ch+*Gl*-HD-2: High-cholesterol diet (0.5%) + *Gl-*2 high dose (1.0%). Each group was fed *ad libitum* for 43 d, including water, according to experimental diets (Table 1). Food consumption was recorded every day, whereas body weight was recorded twice per week. During the last week, feces were collected daily for gut microbiota and bile acid analyses. At the end of the study, mice were deprived of food and water for 8 h before blood sampling, anesthetized with 3% sevoflurane, and then sacrificed on day 43. Blood was collected via the portal vein. Serum was obtained by centrifugation at 1000 g for 10 min and was stored at -70°C until analysis. The liver was rapidly excised, frozen in liquid N_2_, and stored at -70°C. Approval for this experiment was issued by the Animal Research Regulation Committee INCMNSZ at Mexico City (Permit number: FNU-1180-15/16-1).

**Table 1:** Composition of seven tested experimental diets received by C57BL/6 mice groups in this study, according to the standard AIN-93G diet.

Ingredients (g/kg)	Ctrl	Ch	Ch+Sim	Ch+*Gl*-LD-1	Ch+*Gl*-HD-1	Ch+*Gl*-LD-2	Ch+*Gl*-HD-2
**Cystein**	3.0	3.0	3.0	3.0	3.0	3.0	3.0
**Choline**	2.5	2.5	2.5	2.5	2.5	2.5	2.5
**Vitamins**	10.0	10.0	10.0	10.0	10.0	10.0	10.0
**Cellulose**	50.0	50.0	50.0	50.0	50.0	50.0	50.0
**Minerals**	35.0	35.0	35.0	35.0	35.0	35.0	35.0
**Soyabean oil**	70.0	70.0	70.0	70.0	70.0	70.0	70.0
**Starch**	397.5	392.5	392.5	392.5	392.5	392.5	392.5
**Dextrin**	132.0	132.0	132.0	132.0	132.0	132.0	132.0
**Saccharose**	100.0	100.0	100.0	100.0	100.0	100.0	100.0
**Casein**	200.0	200.0	200.0	200.0	200.0	200.0	200.0
**Cholesterol**	-	5.0	5.0	5.0	5.0	5.0	5.0
**Simvastatin**	-	-	0.3	-	-	-	-
***Gl*-1 extract**	-	-	-	5.0	10.0	-	-
***Gl*-2 *extract***	-	-	-	-	-	5.0	10.0

*Gl*: *Ganoderma lucidum*. Ctrl: Control diet. Ch: High cholesterol diet (0.5%). Ch+Sim: High cholesterol diet (0.5%) + simvastatin (0.03 g/100 g). Ch+*Gl*-LD-1: High cholesterol diet (0.5%) + *Gl*-1 low dose (0.5%). Ch+*Gl*-HD-1: High cholesterol diet (0.5%) + *Gl*-1 high dose (1.0%). Ch+*Gl*-LD-2: High cholesterol diet (0.5%) + *Gl*-2 low dose (0.5%). Ch+*Gl*-HD-2: High cholesterol diet (0.5%) + *Gl*-2 high dose (1.0%).

### Serum biochemical parameters

The serum concentrations of total cholesterol (TC), total triglycerides (TG), low-density lipoprotein cholesterol (LDL-C), glucose, alanine transaminase (ALT), and aspartame transaminase (AST) were measured using a COBAS C111 analyzer (Roche Diagnostics Ltd., Switzerland) [17].

### Liver cholesterol and triglyceride concentrations

Liver lipids were extracted from 100 mg of tissue using chloroform-methanol, according to the method described by Folch et al. [18]. The organic layer was dried using liquid nitrogen and solubilized in isopropanol/Triton X-100 (10%). The concentrations of total cholesterol and triglycerides were measured with an enzymatic colorimetric kit (DiaSys Diagnostic Systems, Germany).

### Histological analysis

Liver samples from mice were dissected and immediately fixed with ice-cold paraformaldehyde (4% w/v), dissolved in phosphate buffer, and subsequently dehydrated and embedded in paraffin. Two sections (4-μm) per block were then stained with hematoxylin and eosin [19] and analyzed under a microscope (Leica DM750, Germany).

### Fecal bile acid analysis by gas chromatography (GC)

Stools from C57BL/6 mice were collected during the last week of study, dried, weighed, and ground for measurements of fecal bile acid excretion. Dried fecal samples from each experimental group (100 mg) were mixed in a saline solution, and norcholic acid was added as an internal standard (100 mg/l). Fecal bile acids were extracted [20] and analyzed by GC (Agilent 6850 with flame ionization detector, U.S.A.) using a capillary column (Innowax; J&W Scientific, U.S.A.) as previously described [21].

### RNA extraction

Total RNA was isolated from frozen mouse liver using the TRIzol reagent (Invitrogen, U.S.A.), according to the manufacturer’s instructions. The quantification of RNA was performed using the Nanodrop 2000 spectrophotometer (Thermo Scientific, U.S.A.).

### Quantification of gene expression by reverse transcription polymerase chain reaction (RT-PCR)

RNA was reverse-transcribed to cDNA using Moloney murine leukemia virus (MMLV) reverse transcriptase (Invitrogen). For the real-time PCR analyses of cDNA from the different samples, amplifications were performed using the SYBR Green System in a Light Cycler 4800 thermal cycler system (Roche Diagnostics Ltd., Switzerland). The primers used are described in S2 Table, and beta-2 microglobulin (β2M) was used as a housekeeping gene. Expression values were obtained as the relative expression of the target gene versus the constitutively expressed β2M gene [relative expression = 2 − (Ct, Target gene—Ct, Reference gene)]. Primers for PCR amplification were designed using the Primer3 program (Howard Hughes Medical Institute, U.S.A.). Assays for each gene were performed in triplicate.

### Protein extraction and Western blotting

Liver tissue was homogenized in RIPA buffer containing 1 mmol/l sodium fluoride, 2 mmol/l sodium orthovanadate and complete protease inhibitor cocktail tablets (Roche Applied Science, Mannheim, Germany). Total protein (30 μg) was loaded on 10% polyacrylamide gels, separated by SDS-PAGE and transferred to polyvinylidene difluoride (PVDF) membranes. Blots were blocked with nonfat dry milk (Bio-Rad, Hercules, CA, U.S.A.) and incubated overnight at 4°C with the different primary antibodies, as follows: Fasn (Santa Cruz Biotechnology, sc-20140, 1:18000), Acaca (Millipore, Catalog# 04–332, Lot# 2475628, 1:5000), Srebp1c (Santa Cruz Biotechnology, sc-367, 1:10000), and actin (Santa Cruz Biotechnology, sc-1615, 1:1000), which were all diluted in 4 ml 1x TBS, 5% non-fat milk, and 0.1% Tween 20. Then, the blots were incubated with different secondary antibodies, as follows: anti-rabbit IgG-HRP (Santa Cruz Biotechnology, sc-2004, 1:3000) and anti-goat IgG-HRP (Santa Cruz Biotechnology, sc-2768, 1:3000) diluted in 4 ml 1x TBS, 5% non-fat milk and 0.1% Tween 20, and visualized using a ChemiDoc XRS+ System with Image Lab Software (Bio-Rad, U.S.A.). Bands were analyzed using the ImageJ 1.42p digital imaging processing program (http://rsb.info.nih.gov/ij/March/27/2012).

### Gut microbiota analysis

Fecal samples from mice were immediately collected and frozen at -70°C. DNA extraction was performed using the QIAamp DNA Stool Mini Kit (Qiagen, U.S.A.), according to the manufacturer's instructions. Variable regions 3–4 of the 16S rRNA gene were amplified using specific forward and reverse primers 5´-3´ and 3´-5´ (S3 Table), respectively, containing the Illumina adapter overhang nucleotide sequences. PCR was performed in 25 μl reactions containing 2.5 μl of microbial genomic DNA (5 ng/μl in 10 mM Tris, pH 8.5), 12.5 μl of high-fidelity DNA polymerase 2x KAPA HiFi HotStart ReadyMix, and 5 μl of each primer (1 mM). The temperature cycling protocol consisted of one initial denaturation cycle at 95°C for 3 min, followed by 25 cycles at 95°C for 30 s, 55°C for 30 s, and 72°C for 30 s. Final primer extension was performed at 72°C for 5 min. Ampure XP bits were used to purify the 16S V3-V4 amplicon. Amplicons were quantified on the Agilent 2100 Bioanalyzer (Agilent Technologies, U.S.A.). The amplicon size was approximately 550 bp. Index PCR was then performed to attach dual indices in 50 μl reactions containing 5 μl of first PCR products and 25 μl reactions containing 2.5 μl of microbial genomic DNA (5 ng/μl in 10 mM Tris, pH 8.5), 12.5 μl of the high-fidelity DNA polymerase 2x KAPA HiFi HotStart ReadyMix, and 25 μl of high-fidelity DNA polymerase 2x KAPA HiFi HotStart ReadyMix, and two 5 μl of each Index primer. The Index PCR was performed in a thermal cycler according to the following temperature cycling protocol: one initial cycle at 95°C for 3 min, followed by 8 cycles at 95°C for 30 s, 55°C for 30 s, and 72°C for 30 s. Final primer extension was performed at 72°C for 5 min. Amplicons were cleaned using Ampure XP bits and were quantified on the Agilent 2100 Bioanalyzer. The amplicon size was approximately 610 bp, and the concentration of double-strand DNA was measured using a Qubit 3.0 fluorometer with the highly sensitive kit. The final amplicon library was pooled in equimolar concentrations. Sequencing was performed on the Illumina MiSeq platform (MiSeq Reagent Kit V.3, 600 cycles), according to the manufacturer's instructions, to generate paired-end reads 300 bases in length for each direction. Overlapping paired-end reads were merged using fastq-join and were processed with QIIME V.1.9 [22]. Only Illumina reads with an average score above 20 were retained for further analysis. Reads were chimera checked and assigned to operational taxonomic units (OTUs) using usearch V5.2.236 [23] at a 97% similarity threshold. In this way, 99.9%, 99.7%, 99.6%, 90.4% and 69.7% of the reads were assigned to phylum, class, order, family and genus levels, respectively.

### Statistical analysis

Results are expressed as the means ± SEM. Statistical significance was determined by one-way ANOVA (Bonferroni post-hoc test), as appropriate, using statistical software (2012 SAS Institute Inc., U.S.A.). Differences were considered significant at p < 0.05.

## Results

### Nutrient composition of *Gl* extracts

*Gl*-1 and *Gl*-2 contained carbohydrates (0.58%), glucans (15.96–17.01%, w/w), total protein (0.315–0.365%), total dietary fiber (0.10–0.15%), fat (0.01%), vitamins (B_1_, B_2_, B_3_, B_6_, B_12_, and D), minerals (calcium, copper, ion, magnesium, manganese, phosphorus, potassium, selenium, sodium, and zinc), several organic acids, and an energy content of 4 kcal/100 g extract. There were differences between the nutrient profiles of *Gl*-1 and *Gl*-2, including total protein, total dietary fiber, vitamins (B_1_, B_2_, B_3_, B_9_, B_12_, and D), and minerals (copper, ion, magnesium, manganese, phosphorus, potassium, sodium, and zinc). These differences were originated by the addition of ASA (10 mM) to the substrate, as all other variables remained constant during mushroom cultivation (*i*.*e*., species, strain, substrate, and environmental conditions). Interestingly, there were small differences in the total content of polyphenols and in the antioxidant capacity of both extracts (S1 Table).

### Effects of *Gl* extracts on food intake and mouse body weight

At the beginning of the study, there was no significant difference in body weight among the seven groups (p = 0.244). Daily food intake was 3.45 ± 0.22 g/d averaged over d1-d43 for all groups. The Ch+*Gl*-HD-2 group had a slightly higher food intake (Table 2). This group received a high dose of *Gl*-2 extract obtained from basidiomata cultivated on the substrate treated with ASA (10 mM). There were no significant differences in weight gain among the groups; interestingly, the weight gain tended to be lower in the Ch+*Gl*-HD-2 group than in the rest of the groups.

**Table 2:** Average data from food intake, weight gain, and serum biochemical parameters from C57BL/6 mice studied during 43 days.

Variable	Ctrl	Ch	Ch+Sim	Ch+*Gl*-LD-1	Ch+*Gl*-HD-1	Ch+*Gl*-LD-2	Ch+*Gl*-HD-2	*p*
**Food intake (g/day)**	3.6 ± 0.2	3.1 ± 0.2	3.3 ± 0.2	3.6 ± 0.2	3.5 ± 0.2	3.5 ± 0.2	3.7 ± 0.2	0.244
**Weight gain (g)**	2.2 ± 0.8	2.8 ± 1.3	1.5 ± 1.1	1.8 ± 0.4	2.0 ± 1.2	1.9 ± 0.6	0.5 ± 0.7	0.697
**TC (mg/dL)**	99.1 ± 4.5 ^ab^	110.2 ± 5.5 ^a^	92.5 ± 2.3 ^bc^	81.7 ± 2.5 ^c^	88.8 ± 1.7 ^bc^	89.0 ± 3.4 ^bc^	80.3 ±4.7 ^c^	<0.0001
**TG (mg/dL)**	91.7 ± 9.2 ^a^	86.7 ± 6.6 ^a^	63.1 ± 3.8 ^bc^	46.3 ± 1.6 ^c^	72.6 ± 1.2 ^ab^	55.8 ± 4.1 ^bc^	57.0 ± 3.5 ^bc^	>0.0001
**LDL-C (mg/dL)**	25.8 ± 0.9 ^bc^	35.6 ± 2.7 ^a^	34.8 ± 2.6 ^ab^	29.7 ± 2.4 ^abc^	26.4 ± 1.7 ^abc^	34.0 ± 1.8 ^ab^	23.1 ± 1.1 ^c^	0.0001
**Glucose (mg/dL)**	279.5 ± 12.4	309.2 ± 19.1	304.8 ± 16.5	249.0 ± 11.9	310.7 ± 9.8	292.9 ± 8.0	290.3 ± 16.3	0.0405
**ALT (U/L)**	20.3 ± 1.3 ^abc^	25.3 ± 1.0 ^a^	24.1 ± 0.6 ^ab^	20.0 ± 0.9 ^bc^	18.5 ± 0.5 ^c^	20.4 ± 1.1 ^abc^	19.2 ± 1.6 ^c^	0.0001
**AST (U/L)**	46.7 ± 1.5 ^bc^	61.8 ± 4.0 ^a^	51.6 ± 0.3 ^ab^	40.8 ± 1.7 ^c^	54.1 ± 2.8 ^ab^	52.9 ± 1.9 ^ab^	43.7 ± 1.8 ^bc^	<0.0001

Data are presented as mean ± SEM, n = 8. Means in a row followed by differing superscript letters indicate significant difference, p < 0.05. Values (*p*) correspond to one way ANOVA from the Bonferroni's Multiple Comparison Test. *Gl*: *Ganoderma lucidum*. Ctrl: Control diet. Ch: High cholesterol diet (0.5%). Ch+Sim: High cholesterol diet (0.5%) + simvastatin (0.03 g/100 g). Ch+*Gl*-LD-1: High cholesterol diet (0.5%) + *Gl*-1 low dose (0.5%). Ch+*Gl*-HD-1: High cholesterol diet (0.5%) + *Gl*-1 high dose (1.0%). Ch+*Gl*-LD-2: High cholesterol diet (0.5%) + *Gl*-2 low dose (0.5%). Ch+*Gl*-HD-2: High cholesterol diet (0.5%) + *Gl*-2 high dose (1.0%). TC: Total cholesterol. TG: Triglycerides. LDL-C: Low-density lipoprotein cholesterol. ALT: Alanine transaminase. AST: Aspartate transaminase.

### Effects of *Gl* extracts on mouse serum biochemical parameters

To study the effects of *Gl* extracts on blood lipids, we measured the serum concentrations of TC, LDL-C, and TG. As expected, the consumption of a diet containing 0.5% cholesterol by the Ch group increased serum TC compared to the control group (Ctrl). The addition of simvastatin, a cholesterol-lowering drug, prevented the elevation of serum cholesterol despite the addition of 0.5% of cholesterol to the diet. The mice from the Ch+Sim group had 16.1% less serum TC compared with the Ch group. The addition of *Gl*-1 and *Gl*-2 extracts to the diet significantly reduced serum TC compared with the Ch group, particularly in the Ch+*Gl*-LD-1 (25.8%) and Ch+*Gl*-HD-2 groups (27.1%). The reduction in TC was accompanied by a decrease in the LDL-C concentration, especially in the Ch+*Gl*-HD-2 group (35.1%, p 0.0001). Interestingly, the consumption of either simvastatin or *Gl*-1 and *Gl*-2 extracts decreased TG (p<0.0001). Serum glucose was not modified by dietary treatments. However, the lowest glucose level was recorded in the Ch+*Gl*-LD-1 group. There were significant differences in the serum transaminases ALT and AST in relation to dietary treatments. The serum ALT concentration in the mouse groups fed *Gl* extracts (Ch+*Gl*-LD-1, Ch+*Gl*-HD-1, and Ch+*Gl*-HD-2) was significantly lower (p<0.0001) than that in the Ch group. Likewise, the Ch+*Gl*-LD-1 and Ch+*Gl*-HD-2 groups had significantly lower (p<0.0001) serum AST concentrations than the Ch and Ch+Sim groups (Table 2).

### Effects of *Gl* extracts on hepatic lipids

**Fig 1:**
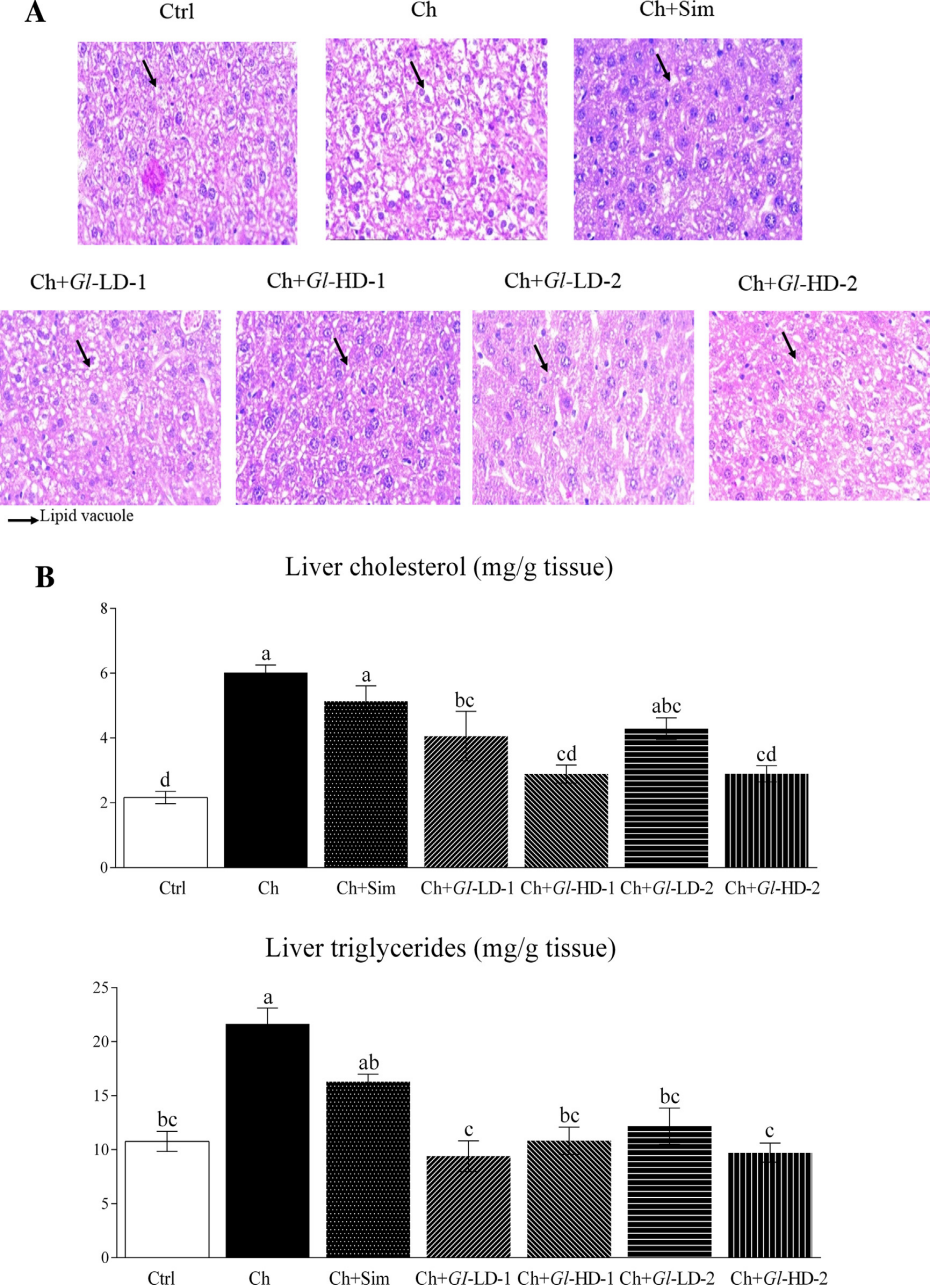
Effect of experimental diets on lipid accumulation in the liver of C57BL/6 mice. A: Hepatic tissue stained with haematoxylin and eosin, showing differences in lipid accumulation among experimental groups. B: Lipid accumulation in the liver assessing levels of cholesterol and triglycerides. Data are presented as mean ± SEM. Means in a column showing differing letters indicate statistically significant difference, p < 0.05. Values correspond to one way ANOVA from the Bonferroni's Multiple Comparison Test. Ctrl: Control diet. Ch: High cholesterol diet (0.5%). Ch+Sim: High cholesterol diet (0.5%) + simvastatin (0.03 g/100 g). Ch+*Gl*-LD-1: High cholesterol diet (0.5%) + *Gl*-1 extract, low dose (0.5%). Ch+*Gl*-HD-1: High cholesterol diet (0.5%) + *Gl*-1 extract, high dose (1.0%). Ch+*Gl*-LD-2: High cholesterol diet (0.5%) + *Gl*-2 extract, low dose (0.5%). Ch+*Gl*-HD-2: High cholesterol diet (0.5%) + *Gl*-2 extract, high dose (1.0%).

Because we observed a reduction in the concentrations of circulating blood lipids, we performed a histological analysis of liver sections from mice with all the dietary treatments. The analysis revealed that the consumption of a high-cholesterol (0.5%) diet for 43 d increased the number of lipid vacuoles in the liver (Fig 1A). The consumption of simvastatin reduced the number of lipid vacuoles. The consumption of low and high doses of *Gl*-1 and *Gl*-2 extracts almost prevented the accumulation of lipids in the liver. Analyses of hepatic cholesterol and triglycerides confirmed the histological analysis. The consumption of simvastatin reduced the concentration of hepatic triglycerides, which was associated with a reduction in the concentration of hepatic cholesterol. There was a significant reduction in cholesterol in the livers of mice fed high doses of *Gl* extracts (Ch+*Gl*-HD-1 and Ch+*Gl*-HD-2), including one group fed a low dose of *Gl*-1 extract (Ch+*Gl*-LD1), compared with the Ch group (p<0.001), which received a high-cholesterol diet only. The consumption of *Gl*-1 and *Gl*-2 extracts at high doses effectively reduced hepatic cholesterol by 51.9%. Both *Gl* extracts were also capable of reducing hepatic triglycerides (p<0.001) between 43.8% and 56.6% (Fig 1B).

### Effects of *Gl* extracts on the expression of lipogenic genes and Hmgcr

**Fig 2:**
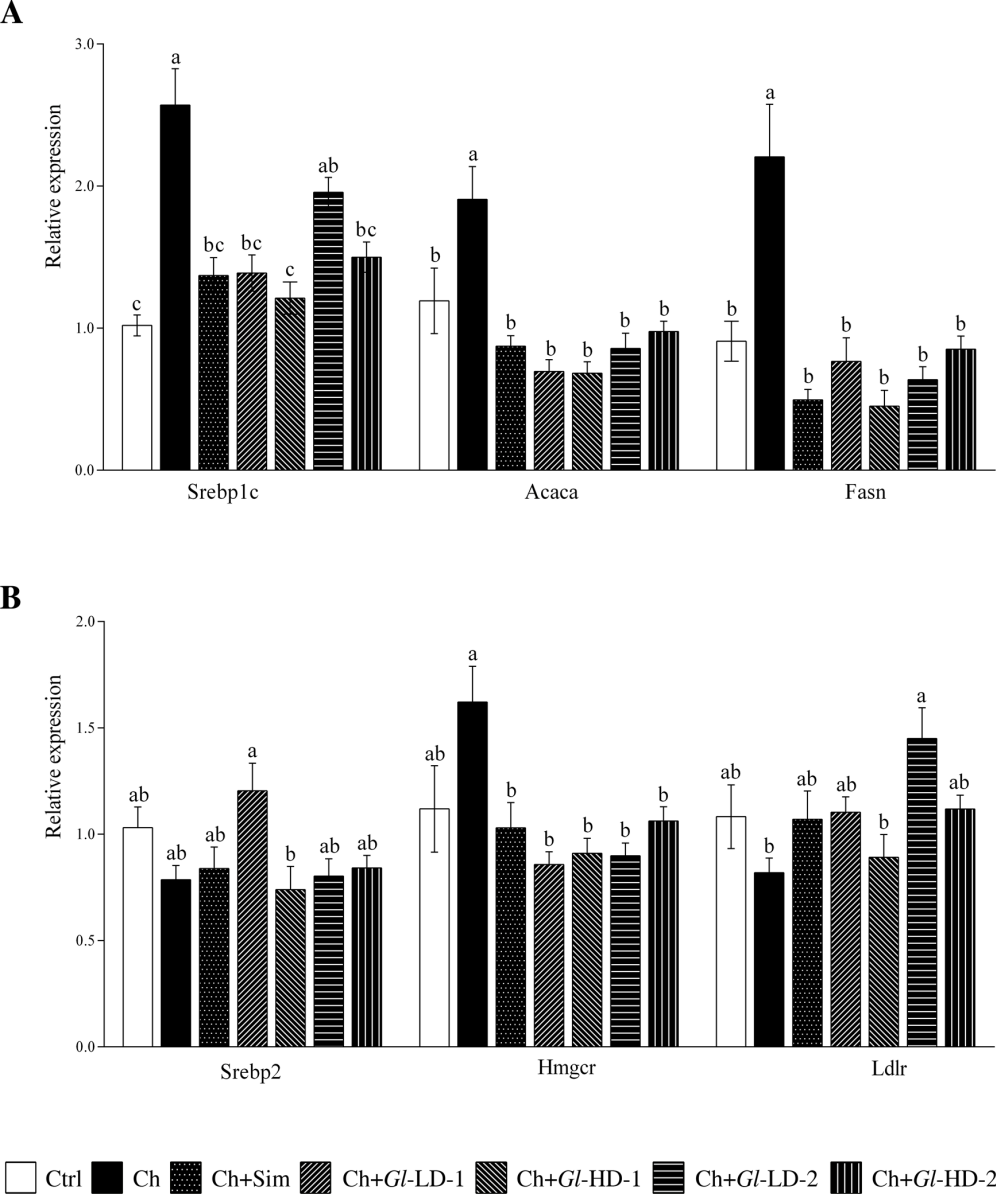
Effect of experimental diets on the expression of genes related to lipogenic processes in the liver of C57BL/6 mice. A: Lipogenic genes. B: Genes associated to cholesterol metabolism. Data are presented as mean ± SEM. Means in a column showing differing letters indicate statistically significant difference, p < 0.05. Values correspond to one way ANOVA from the Bonferroni's Multiple Comparison Test. Ctrl: Control diet. Ch: High cholesterol diet (0.5%). Ch+Sim: High cholesterol diet (0.5%) + simvastatin (0.03 g/100 g). Ch+*Gl*-LD-1: High cholesterol diet (0.5%) + *Gl*-1 extract, low dose (0.5%). Ch+*Gl*-HD-1: High cholesterol diet (0.5%) + *Gl*-1 extract, high dose (1.0%). Ch+*Gl*-LD-2: High cholesterol diet (0.5%) + *Gl*-2 extract, low dose (0.5%). Ch+*Gl*-HD-2: High cholesterol diet (0.5%) + *Gl*-2 extract, high dose (1.0%).

**Fig 3:**
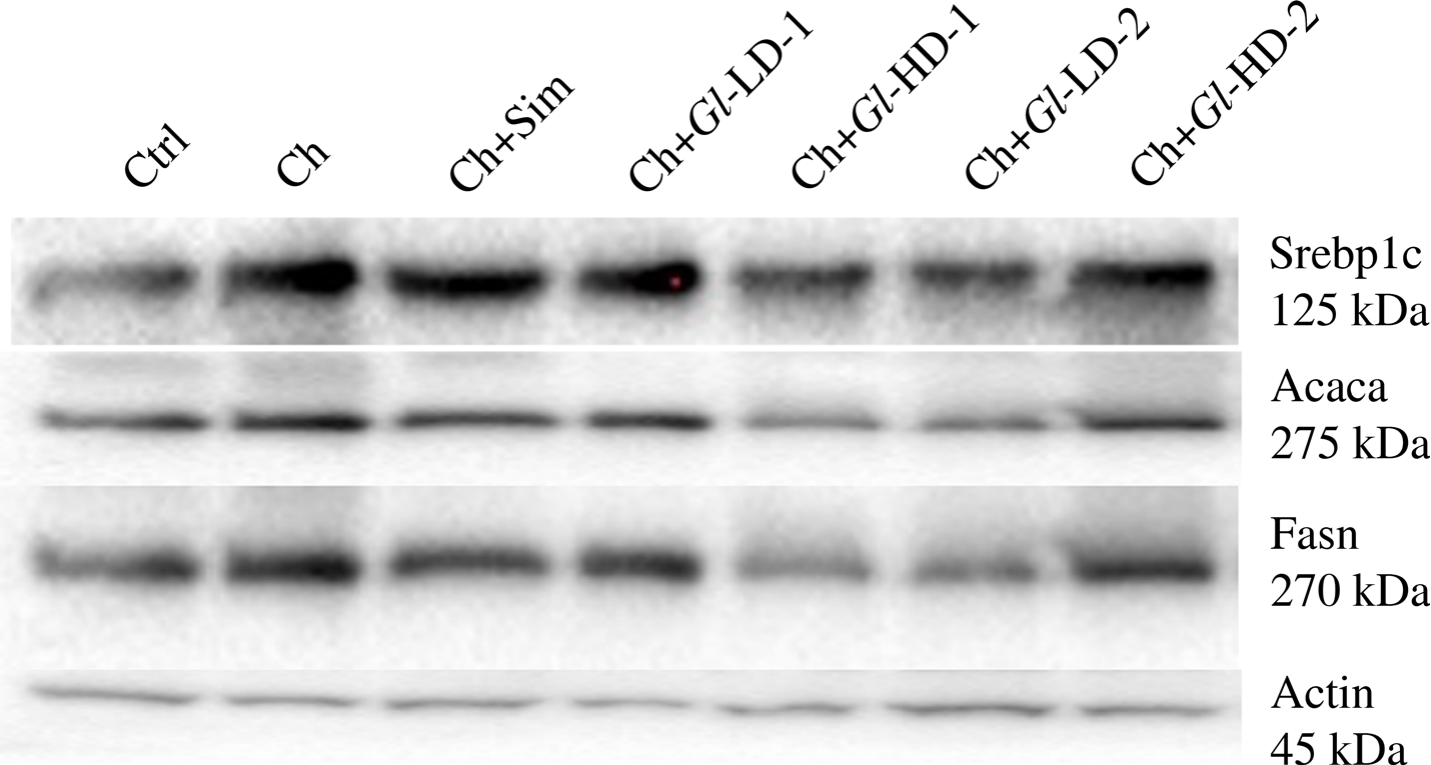
Abundance of proteins involved in the lipogenesis process in the liver (Srebp1c, Acaca and Fasn/Actin). Ctrl: Control diet. Ch: High cholesterol diet (0.5%). Ch+Sim: High cholesterol diet (0.5%) + simvastatin (0.03 g/100 g). Ch+*Gl*-LD-1: High cholesterol diet (0.5%) + *Gl*-1 extract, low dose (0.5%). Ch+*Gl*-HD-1: High cholesterol diet (0.5%) + *Gl*-1 extract, high dose (1.0%). Ch+*Gl*-LD-2: High cholesterol diet (0.5%) + *Gl*-2 extract, low dose (0.5%). Ch+*Gl*-HD-2: High cholesterol diet (0.5%) + *Gl*-2 extract, high dose (1.0%).

To understand the mechanism of action of *Gl* extracts in the reduction of hepatic lipids, the RNA and protein abundance of key lipogenic genes were assessed, as well as the RNA abundance of genes associated to cholesterol metabolism (Figs 2 and 3). We first measured the RNA abundance of the key lipogenic genes Srebp1c, Acaca and Fasn. The results clearly showed that the addition of *Gl*-1 and *Gl*-2 extracts to the diet significantly reduced the expression of these three genes (Fig 2A), establishing a direct effect of both extracts on reducing hepatic fatty acid synthesis and, hence, hepatic triglyceride accumulation. Similarly, protein abundance of Srebp1c, Acaca and Fasn was lower in the groups fed with both *Gl* extracts. *Gl*-1 and *Gl*-2 extracts modulated the expression of genes associated with cholesterol metabolism, particularly Srebp2, Hmgcr and Ldlr, which showed relative expression levels similar to those of the Ctrl group. Hmgcr is the rate-limiting enzyme in cholesterol biosynthesis. In fact, the consumption of simvastatin, an inhibitor of this enzyme, and *Gl-1* and *Gl-2* extracts significantly reduced (p<0.001) the mRNA abundance of Hmgcr by 34.6% to 46.9% (p<0.001) (Fig 2B). It was also observed that the low dose of the *Gl*-2 extract (Ch+*Gl*+LD-2) significantly increased the expression of the Ldlr gene compared to the Ctrl and Ch (p = 0.009) groups (Fig 2B).

### Effects of *Gl* extracts on the expression of genes involved in cholesterol transport and bile acid synthesis

**Fig 4:**
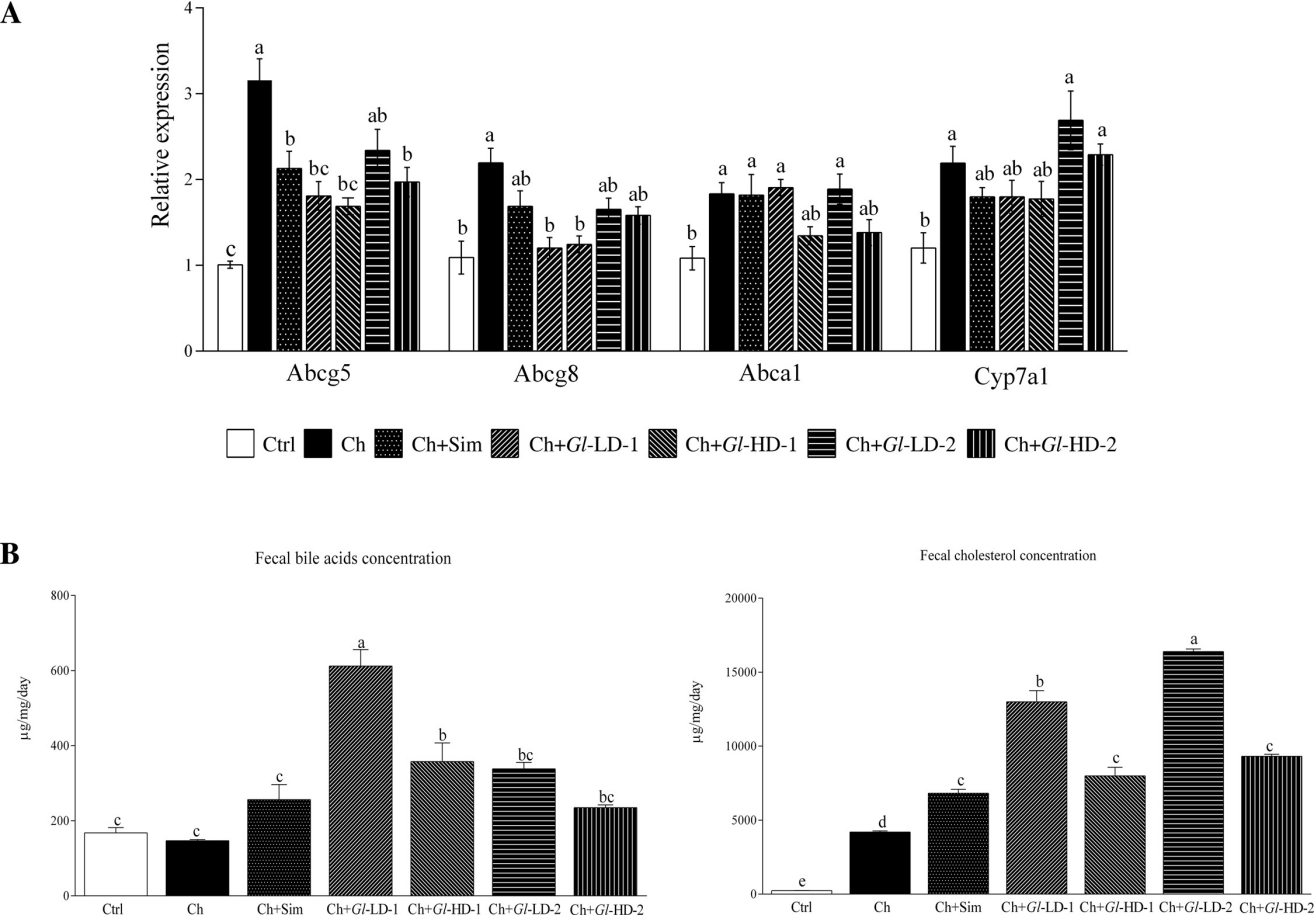
Effect of the experimental diets on C57BL/6 mice. A: Expression of genes related to reverse cholesterol transport in the liver. B: Amount of bile acids and cholesterol in feces. Data are presented as mean ± SEM. Means in a column showing differing letters indicate statistically significant difference, p < 0.05. Values correspond to one way ANOVA from the Bonferroni's Multiple Comparison Test. Ctrl: Control diet. Ch: High cholesterol diet (0.5%). Ch+Sim: High cholesterol diet (0.5%) + simvastatin (0.03 g/100 g). Ch+*Gl*-LD-1: High cholesterol diet (0.5%) + *Gl*-1 extract, low dose (0.5%). Ch+*Gl*-HD-1: High cholesterol diet (0.5%) + *Gl*-1 extract, high dose (1.0%). Ch+*Gl*-LD-2: High cholesterol diet (0.5%) + *Gl*-2 extract, low dose (0.5%). Ch+*Gl*-HD-2: High cholesterol diet (0.5%) + *Gl*-2 extract, high dose (1.0%).

We first measured the liver expression of genes involved in reverse cholesterol transport. The data demonstrated that the consumption of cholesterol significantly elevated the expression of the Abcg5/g8 genes by 2.1- and 1.0-fold, respectively (Fig 4A). The addition of simvastatin and the *Gl*-2 extract also increased the expression of these genes, albeit to a lesser extent. The *Gl*-1 extract did not stimulate the expression of these genes, particularly Abcg8. The expression of Abca1 was also increased by cholesterol; however, none of the extracts further stimulated the expression of this transporter. Interestingly, the expression of Cyp7a1 was also increased by the inclusion of cholesterol in the diet, but it was not further increased by any of the *Gl* extracts. Furthermore, the consumption of the *Gl* extracts significantly increased the amount of fecal bile acids, particularly the *Gl*-1 extract at both low and high doses. Surprisingly, mice fed the low dose of *Gl*-1 extract excreted 41.6% more fecal bile acids than those that consumed the high dose (Fig 4B). Interestingly, the mouse group fed a high-cholesterol diet containing simvastatin (Ch+Sim) and the groups fed *Gl*-1 and *Gl*-2 extracts (Ch+Gl-LD-1, Ch+*Gl*-HD-1, Ch+*Gl*-LD-2, and Ch+*Gl*-HD-2) all excreted significantly more fecal cholesterol than the high-cholesterol (Ch) group. Mice fed the low dose of both *Gl* extracts excreted significantly more fecal cholesterol than the other groups (p<0.001) (Fig 4B).

### The effects of *Gl* extracts on the gut microbiota

**Fig 5:**
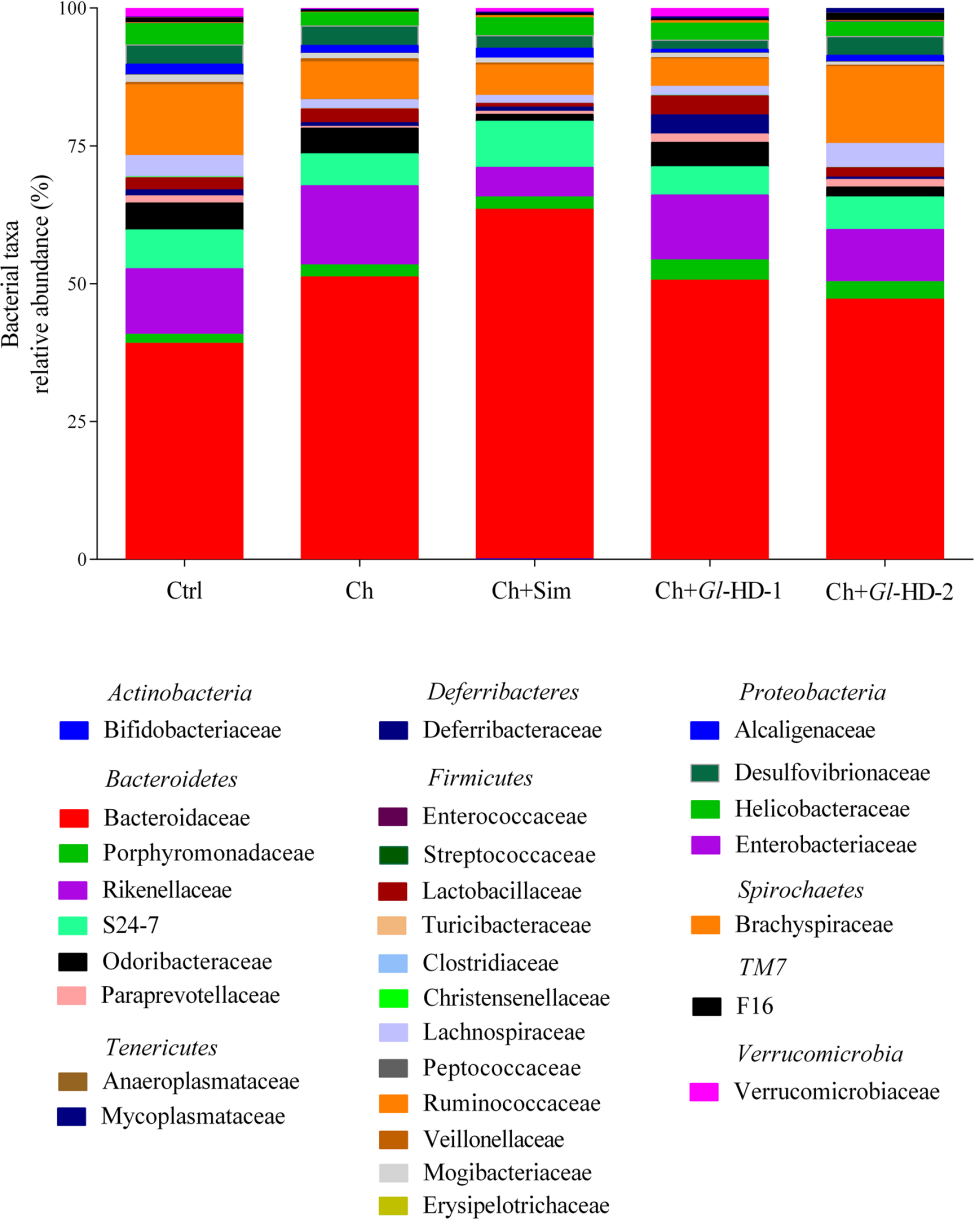
Effect of experimental diets on the gut microbiota from five different groups of C57BL/6 mice. Relative abundance at the bacterial family level. Ctrl: Control diet. Ch: High cholesterol diet (0.5%). Ch+Sim: High cholesterol diet (0.5%) + simvastatin (0.03 g/100 g). Ch+*Gl*-HD-1: High cholesterol diet (0.5%) + *Gl*-1 extract, high dose (1.0%). Ch+*Gl*-HD-2: High cholesterol diet (0.5%) + *Gl*-2 extract, high dose (1.0%).

**Fig 6:**
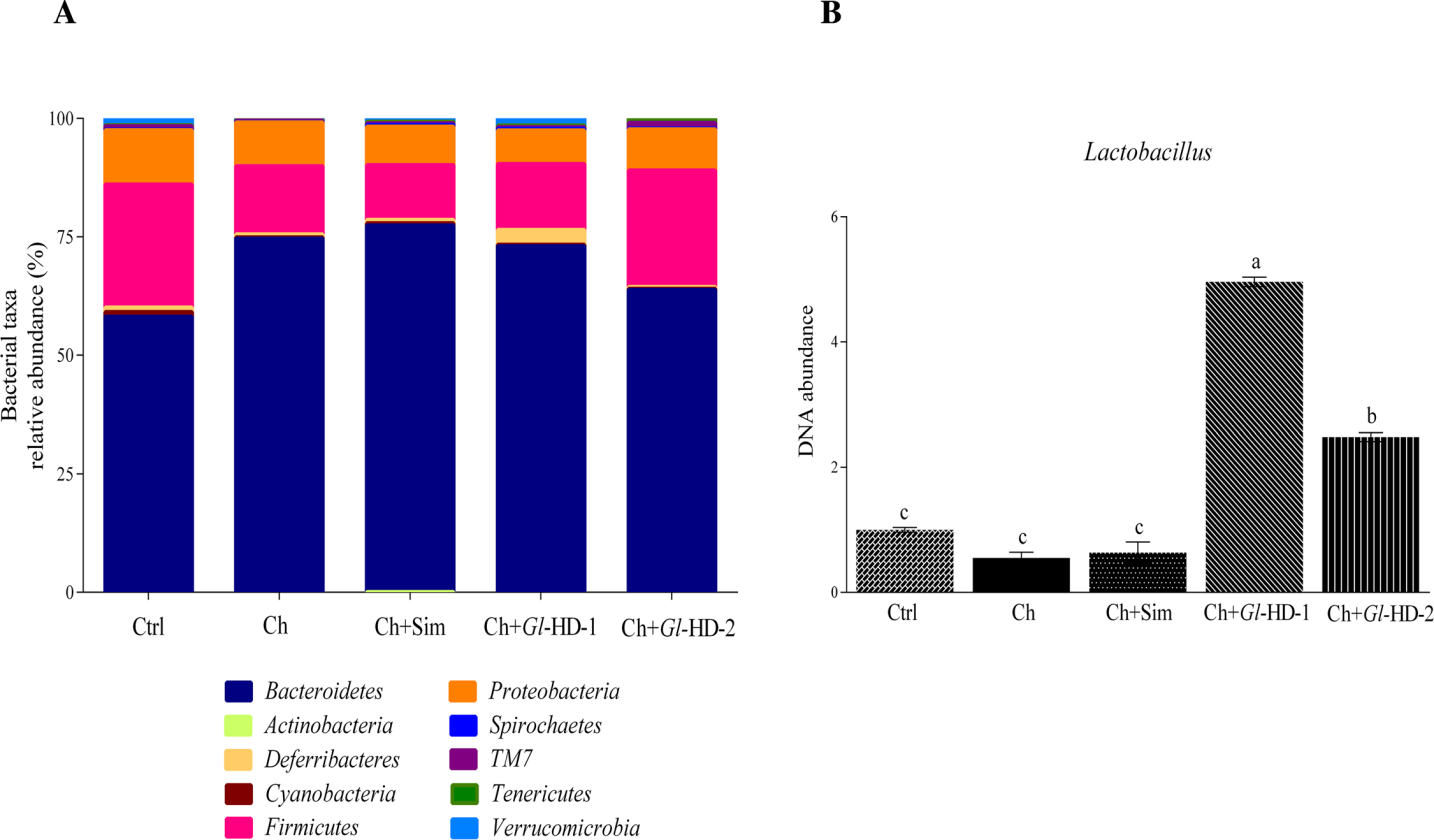
Effect of experimental diets on the gut microbiota from five different groups of C57BL/6 mice. A: Relative abundance at the bacterial *phyla* level. B: DNA abundance of *Lactobacillus* by RT-PCR. Ctrl: Control diet. Data are presented as mean ± SEM. Means in a column showing differing letters indicate statistically significant difference, p < 0.05. Values correspond to one way ANOVA from the Bonferroni's Multiple Comparison Test. Ch: High cholesterol diet (0.5%). Ch+Sim: High cholesterol diet (0.5%) + simvastatin (0.03 g/100 g). Ch+*Gl*-HD-1: High cholesterol diet (0.5%) + *Gl*-1 extract, high dose (1.0%). Ch+*Gl*-HD-2: High cholesterol diet (0.5%) + *Gl*-2 extract, high dose (1.0%).

**Fig 7:**
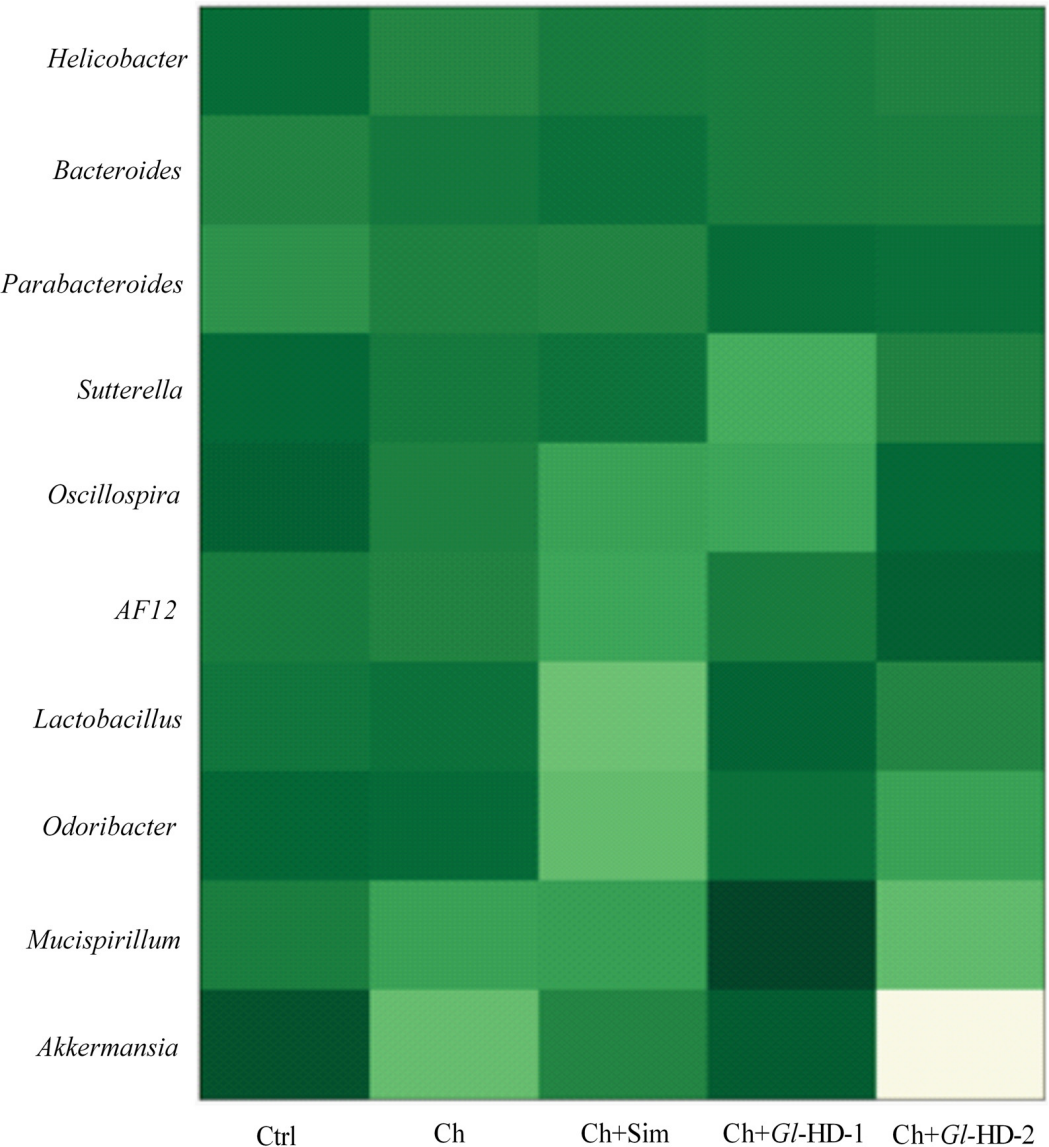
A heatmap showing the relative abundance of bacterial genera, which cover 95% of all reads assigned to this level in mice groups studied. Ctrl: Control diet. Ch: High cholesterol diet (0.5%). Ch+Sim: High cholesterol diet (0.5%) + simvastatin (0.03 g/100 g). Ch+*Gl*-HD-1: High cholesterol diet (0.5%) + *Gl*-1 extract, high dose (1.0%). Ch+*Gl*-HD-2: High cholesterol diet (0.5%) + *Gl*-2 extract, high dose (1.0%).

The gut microbiota was assessed in stool samples from mice receiving different high-cholesterol diets, as shown in Figs 5–7. Sample sequencing from mouse groups consuming high doses of *Gl*-1 and *Gl*-2 extracts (Ch+*Gl*-HD-1 and Ch+*Gl*-HD-2) resulted in >100,000 reads, with *Bacteroidetes*, *Firmicutes*, and *Proteobacteria* representing *ca*. 95% of all sequences at the phylum level. The rest of the sequences belonged to *Deferribacteres*, *Verrucomicrobia*, *Cyanobacteria*, *TM7*, *Tenericutes*, *Spirochaetes*, and *Actinobacteria* (Fig 6). Interestingly, at the phyla level, the addition of cholesterol to the diet increased the proportion of *Bacteroidetes*, particularly in the Ch (74.6%), Ch+Sim (77.3%), and Ch+*Gl*-HD-1 groups (72.9%) compared to the Ctrl group (58.1%). The mouse group fed a diet containing a high dose of *Gl*-2 extract showed a proportion of *Bacteroidetes* (63.7%) similar to the Ctrl group (Fig 6A). The increase in *Bacteroidetes* with the addition of cholesterol to the diet was accompanied by a reduction in *Firmicutes*. The phylum *Verrucomicrobia* represented 1.05% of the total sequences, with the Ch+*Gl*-HD-1 group showing the greatest abundance (1.48%), followed by the Ctrl group (1.42%). Analysis at the family level indicated that the increase in *Bacteroidetes* was mainly associated with a greater proportion of the *Bacteroidaceae* family. It is important to note that, despite the decrease in *Firmicutes* caused by adding cholesterol to the diet, mice fed a high dose of *Gl*-1 extract (Ch+*Gl*-HD-1) showed an increase in the *Lactobacillaceae* family (3.4%) compared to the Ctrl group (2.2%), whereas the consumption of simvastatin decreased the proportion of the *Lactobacillaceae* family (0.7%; Ch+Sim). At the genus level, the increase in the *Lactobacillaceae* family in the Ch+*Gl*-HD-1 group showed a greater proportion of *Lactobacillus* (4.3%) compared to the Ctrl group (3.0%), whereas simvastatin consumption reduced that proportion to 0.8%. The RT-PCR analysis also demonstrated that the consumption of the *Gl*-1 extract significantly increased the abundance of *Lactobacillus* in the gut microbiota of the Ch+*Gl*-HD-1 group (Fig 6B). A heat map revealed that consumption of the *Gl*-1 extract also increased the genera *Mucispirillum* and *Parabacteroides*, but decreased *Helicobacter*, *Sutterella*, and *Oscillospira*. No strong effect was observed on *Bacteroides*, *AF12*, *Odoribacter*, or *Akkermansia* compared with the control (Fig 7). By contrast, the consumption of *Gl*-2 extract only increased the genera *Parabacteroides* and *AF12*, whereas *Helicobacter*, *Sutterella*, *Odoribacter*, *Mucispirillum*, and *Akkermansia* were reduced compared with the control. In this case, no strong effect was noted on *Bacteroides* or *Oscillospira* (Fig 7).

## Discussion

Hypercholesterolemia is an important health problem worldwide. In the most recent national health survey in Mexico, approximately 13% of the population above 20 years old had elevated serum cholesterol [24]. In the present work, we have shown that the consumption of standardized *Gl*-1 and *Gl*-2 extracts of *Ganoderma lucidum*, derived from basidiomata of Mexican genetic resources cultivated under controlled conditions, represents a promising alternative for the treatment of hypercholesterolemia. Our evidences indicate that either the amount of glucans or the presence of several previously reported bioactive compounds can regulate different mechanisms associated with cholesterol metabolism.

Our data from mice fed a high-cholesterol diet demonstrated that *Gl* extracts maintained low total cholesterol and LDL-C levels below the concentrations observed in mice fed a high-cholesterol diet only. In addition, the consumption of these extracts reduced serum cholesterol levels to an extent similar to that observed when mice were treated with simvastatin, a well-known hypocholesterolemic drug. Similar to statins, *Gl*-1 and *Gl*-2 extracts were capable of decreasing the expression of Hmgcr, the rate-limiting enzyme in cholesterol synthesis. However, previous work did not find lovastatin in the organic or aqueous phases of *G*. *lucidum* [25], suggesting that a compound(s) other than statins is involved. Several other mushroom compounds have also been reported to have positive effects on lipid metabolism, such as lovastatin from *Pleurotus* spp., eritadenine from *Lentinula edodes*, chitosan from *Agaricus bisporus*, and polysaccharides from *Tremella fuciformis* [11, 13, 14, 25–27]. There is evidence that *G*. *lucidum* contains, among other bioactive compounds, 26-oxygenosterols of ganoderol, ganoderal, and ganoderic acid, which downregulate cholesterol biosynthesis by inhibiting the enzyme lanosterol 14α-demethylase that catalyzes the biotransformation of 24,25-dihydrolanosterol to cholesterol. [14]. Further chemical studies are needed to identify novel bioactive compounds associated with lipid metabolism in *Gl*-1 and *Gl*-2 extracts.

Although it is known that the expression of several of the enzymes and proteins involved in the biosynthesis and uptake of cholesterol is controlled by the transcription factor Srebp2, we did not observe significant differences in the expression of this transcription factor. Nonetheless, our results showed upregulation of Ldlr in mice that consumed the *Gl* extracts. Thus, it is necessary to conduct specific studies to determine how *G*. *lucidum* can downregulate Hmgcr. The decrease in the expression of Hmgcr suggests a reduction in the biosynthetic capacity of cholesterol by *G*. *lucidum* that may reduce hepatic cholesterol concentrations. In fact, our results clearly established that *Gl* extracts significantly reduced the hepatic concentrations of both cholesterol and triglycerides, which virtually resulted in the abolishment of lipid droplets in the liver. To enhance the removal of cholesterol from the liver, we observed that *Gl* extracts were capable of upregulating the expression of transporters involved in reverse cholesterol transport, such as Abcg5 and Abcg8, and the biosynthesis of bile acids. Interestingly, most of these genes are regulated by the transcription factor LXR-α, whose activity is stimulated by oxysterols [5]. Although it has not been established whether the oxygenated forms of ganoderic acid can function as potential ligands of LXR-α, there is a possibility that these bioactive compounds may serve as stimulators of LXR-α, thus upregulating the bile acid biosynthesis pathway. Our results showed that *Gl* extracts increased the excretion of fecal bile acids and the concentration of fecal cholesterol compared to the control group. At the present time, we do not have a mechanistic explanation for why the low dose of the *Gl* extracts was more potent at increasing fecal cholesterol excretion.

At present, several compounds associated with health benefits have been identified in standardized *Gl*-1 and *Gl*-2 extracts of *G*. *lucidum*, including the following: α-glucans (*Gl*-1: 14.19% w/w, *Gl*-2: 15.14% w/w); β-glucans (*Gl*-1: 1.77% w/w, *Gl*-2: 1.87% w/w); total dietary fiber (*Gl*-1: 0.15%, *Gl*-2: 0.10%); total polyphenols (*Gl*-1: 2.185% mg/ml GAE, *Gl*-2: 1.858 mg GAE/g); and molecules with high antioxidant capacity that are able to trap reactive oxygen species (*Gl*-1: 350,811.50 μmol of trolox equivalent/g, *Gl*-2: 376,117.06 μmol of trolox equivalent/g). Previous studies also support the important role of the bioactive compounds identified in *Gl*-1 and *Gl*-2 extracts in the present study. For instance, β-glucans from yeast and *Pleurotus ostreatus* significantly lowered plasma cholesterol in mice, rats, and in clinical trials [28–30], and β-glucans from *Trametes versicolor* significantly decreased LDL-C in individuals with hyperlipidemia [26]. In general, the hypocholesterolemic effects, including the lowering of plasma triglycerides, and hepatic total cholesterol and triglycerides, of edible mushrooms (*e*.*g*., *Auricularia auricula*, *Agaricus bisporus*, *P*. *ostreatus*, and *Tremella fuciformis*) have been mainly attributed to dietary fiber [11,31]. In this study, the presence of α-glucans in *G*. *lucidum*, a major component of *Gl*-1 (88.9%) and *Gl*-2 (89.0%) extracts, suggests that these compounds can also synergistically promote the cholesterol-lowering effect of *Gl* extracts. The low solubility and viscosity of β-glucans in the gut have been proposed as a mechanism of action resulting in the decreased absorption of cholesterol and triglycerides, as well as the greater excretion of fecal bile acids and cholesterol [32]. By contrast, the mouse group fed a high-cholesterol diet (Ch) without the inclusion of *Gl* extracts in the diet excreted smaller amounts of bile acids and cholesterol, resulting in significantly greater cholesterol accumulation in the liver and serum.

In addition, antioxidant properties identified in *Gl*-1 and *Gl*-2 extracts may play an important role in mouse lipid metabolism. The antioxidant properties of *Gl*-1 and *Gl*-2 extracts are in part associated with the presence of polyphenols. A clinical trial by Wachtel-Galor *et al*. found an association between the antioxidant capacity of *Ganoderma* and a trend toward improved plasma lipid profiles [33]. Similar hypocholesterolemic effects associated with the inhibition of lipid peroxidation have also been reported for *Lentinula edodes* (polysaccharides), *P*. *citrinopileatus* (extracts), and *P*. *ostreatus* (dried basidiomata) in rats and rabbits [27]. The antioxidant properties of edible mushrooms prevent atherogenesis by decreasing the oxidation of low-density lipoproteins and lipid peroxidation [11].

An additional mechanism by which *Gl* extracts prevent hypercholesterolemia may be associated with the presence of specific microorganisms in the gut microbiota. Several mechanisms have been proposed by which gut microbiota regulates cholesterol metabolism, including the findings that some bacteria can affect enterohepatic circulation, the *de novo* synthesis of bile acids, emulsification, and cholesterol absorption [7] and re-absorption in the intestine [34]. Both in rodents and humans, *Lactobacillus* has the capacity to reduce blood cholesterol levels, possibly as a result of the action of the bacterial enzyme bile salt hydrolase, which can affect cholesterol re-absorption in the intestine [34]. Interestingly, in our study, the analysis of gut microbiota by sequencing of the 16S RNA demonstrated that at the genus level, the addition of *Gl* extracts significantly increased *Lactobacillus*, suggesting that the presence of these microorganisms may also be involved in the reduction of serum cholesterol [35]. The increase in the proportion of *Lactobacillus* and possibly other genera, such as *Parabacteroides* and *Mucispirillum*, may be associated with the prebiotic capacity of the glucans present in *Gl* extracts to increase their abundance. This is in accordance with a previous study that showed that β-glucans stimulated the growth of *Lactobacillus* [36]. In addition, Chang *et al*. [37] recently reported prebiotic effects of Chinese *G*. *lucidum* in mice fed a high-fat diet, using high-molecular-weight polysaccharides isolated from water extracts of mycelium instead of the basidiocarp. However, they did not find an increase in *Lactobacillus* abundance.

The magnitude of biological effects differed between *Gl-1* and *Gl-2* extracts according to their dose. This evidence of detectable differences between *Gl*-1 and *Gl*-2 extracts clearly showed that the addition of acetylsalicylic acid (ASA, 10 mM) to the cultivation substrate modulates the functional properties of *G*. *lucidum*. The ASA induced changes in the nutrient and chemical composition of *Gl*-1 and *Gl*-2 extracts obtained from mature basidiomata, thereby achieving differing bioactivities. The *Gl*1 extract showed better effects on lipid metabolism at low dose, whereas the *Gl*2 extract had some similar effects but at high dose. Thus, despite the small differences in composition between *Gl*-1 and *Gl*-2 extracts, it is important to standardize how to prepare mushroom extracts to obtain specific health benefits.

In summary, this study demonstrates that the consumption of *Gl* extracts can be used as a strategy to control serum cholesterol concentration. It is important to emphasize that the substrate formulation for mushroom cultivation can change its biological properties as we observed in the present study. Standardized and high quality mushroom products are therefore required for the treatment of hypercholesterolemia. Further clinical randomized trials are also needed to support the use of *G*. *lucidum*, as no adverse effects were recorded in this study. It is important to consider the dose-response in humans and the length of the study to confirm reductions in plasma cholesterol concentration in subjects with different levels of hypercholesterolemia. Finally, it is particularly relevant to investigate the possible contribution of including native food resources as a part of dietary strategies for the treatment and prevention of CVD in countries with a high prevalence and elevated rates of morbi-mortality associated with elevated serum cholesterol concentrations.

## Supporting Information

S1 Fig Comparative direct visualization of extracts of *Ganoderma lucidum* in a spectrophotometer (Epoch, Biotek, U.S.A.; wavelength range: 200–1000 nm) using microplates (n = 3). *Gl*-1: Standardized extract from basidiomata cultivated on the control oak sawdust substrate. *Gl*-2: Standardized extract from basidiomata cultivated on oak sawdust substrate treated with acetylsalicylic acid (ASA, 10 mM).
(DOCX)

S1 Table Nutrient composition and analysis of extracts from mature basidiomata of Mexican *Ganoderma lucidum*, cultivated on *Quercus* sawdust (*Gl*-1) and *Quercus* sawdust plus acetylsalicylic acid (10 mM; *Gl*-2). (DOCX)

S2 Table Primer sequences used for reverse transcription polymerase chain reaction (RT-PCR). (DOCX)

S3 Table The 16S rRNA gene-targeted primers used for microbiota analysis in the genus *Lactobacillus*. (DOCX)
