# Supplementary material for: Hypocholesterolemic Properties and Prebiotic Effects of Mexican Ganoderma lucidum in C57BL/6 Mice
Source: PLoS One. 2016 Jul 20;11(7):e0159631. doi: 10.1371/journal.pone.0159631 (PMC4954724; doi:10.1371/journal.pone.0159631)
Supplement: S1 Fig — Gl-1: Standardized extract from basidiomata cultivated on the control oak sawdust substrate. Gl-2: Standardized extract from basidiomata cultivated on oak sawdust substrate treated with acetylsalicylic acid (ASA, 10 mM). (DOCX) [file pone.0159631.s001.docx]

*Gl*-1

*Gl*-2

**Supplementary Fig. 1. Comparative direct visualization of extracts from *Ganoderma lucidum* in a spectrophotometer (Epoch, Biotek, U.S.A.; wavelength range: 200-1000 nm) using microplates (n= 3).** *Gl*-1: Standardized extract from basidiomata cultivated on the control oak sawdust substrate. *Gl*-2: Standardized extract from basidiomata cultivated on oak sawdust substrate treated with acetylsalicylic acid (ASA, 10 mM).
