# Supplementary material for: Hypocholesterolemic Properties and Prebiotic Effects of Mexican Ganoderma lucidum in C57BL/6 Mice
Source: PLoS One. 2016 Jul 20;11(7):e0159631. doi: 10.1371/journal.pone.0159631 (PMC4954724; doi:10.1371/journal.pone.0159631)
Supplement: S1 Table — (DOCX) [file pone.0159631.s002.docx]

**Supplementary Table 1. Chemical composition and analysis of extracts from mature basidiomata of Mexican *Ganoderma lucidum*, cultivated on *Quercus* sawdust (*Gl*-1) and *Quercus* sawdust plus acetylsalicylic acid (10 mM; *Gl*-2).**

| **Analysis** | ***Gl*-1** | ***Gl*-2** | **Unit** | **Method** | **Technique** |
| --- | --- | --- | --- | --- | --- |
| **Total protein** | 0.365 | 0.315 | % | AOAC. 1995. No. 991.20 | Kjeldahl method |
| **Carbohydrates by difference^a^** | 0.58 | 0.58 | % | Merrill & Watt. 1973 | - |
| **Glucose** | 0.1 | 0.1 | % | AOAC. 1995.  No. 996.04 | Ion chromatography |
| **Total sugars** | 0.1 | 0.1 | % | AOAC. 1995.  No. 996.04 | Ion chromatography |
| **Calories^a^** | 4 | 4 | /100 g | Methods of Analysis for Nutritional Labelling, AOAC International. 1993 | - |
| **Total dietary fiber** | 0.15 | 0.10 | % | AOAC. 2003.  No. 985.29 | Gravimetric analysis |
| **Total glucans** | 15.96 | 17.01 | % w/w | AOAC. No. 995.16 | Colorimetric method |
| **α-glucans** | 14.19 | 15.14 | % w/w | AOAC. No. 995.16 | Colorimetric method |
| **β-glucans** | 1.77 | 1.87 | % w/w | AOAC. No. 995.16 | Colorimetric method |
| **Total polyphenols** | 2.185 | 1.858 | mg GAE/g | Folin Ciocalteau assay | Colorimetric method |
| **Reactive species** | 350,811.50 | 376,117.06 | μmol of Trolox Equivalent/g | Oxygen radical absorbance capacity (ORAC) assay | Fluorescence assay |
| **Fat** | 0.01 | 0.01 | % | AOAC. 2000. 989.05 | Gravimetric analysis |
| **Ash** | 0.10 | 0.10 | % | AOAC. 2000.  No. 942.05 | Gravimetric analysis |
| **Vitamin B1 (thiamine)** | 0.03 | 0.025 | mg/100 g | R-Biopharm. 2011. VitaFast Vitamin B1 | Microbiological assay |
| **Vitamin B2 (riboflavin)** | 0.04 | 0.035 | mg/100 g | R-Biopharm. 2011. VitaFast Vitamin B2 | Microbiological assay |
| **Vitamin B3 (niacin)** | 0.47 | 0.42 | mg/100 g | R-Biopharm. 2011. VitaFast Vitamin B3 | Microbiological assay |
| **Vitamin B6 (pyridoxin)** | 0.01 | 0.01 | mg/100 g | R-Biopharm. 2011. VitaFast Vitamin B6 | Microbiological assay |
| **Vitamin B12 (cyanocobalamin)** | 0.05 | 0.045 | µg/100 g | R-Biopharm. 2011. VitaFast Vitamin B12 | Microbiological assay |
| **Vitamin D** | <2.0 | <2.0 | IU/100 g | AOAC. 2009.  No. 982.29 | HPLC |
| **Calcium** | 1.15 | 1.10 | mg/100 g | AOAC. 2005.  No. 974.14 | Atomic absorption spectrometry |
| **Copper** | 120 | 115 | Ppb | Method CFAN/ORS/DBC/CHCB. FDA 2011 | ICP-MS |
| **Iron** | 200 | 335 | Ppb | Method CFAN/ORS/DBC/CHCB. FDA 2011 | ICP-MS |
| **Magnesium** | 2.35 | 2.25 | mg/100 g | AOAC. 2005.  No. 974.14 | Atomic absorption spectrometry |
| **Manganese** | 120 | 135 | Ppb | Method CFAN/ORS/DBC/CHCB. FDA 2011 | ICP-MS |
| **Phosphorus** | 10.5 | 12.5 | mg/100 g | AOAC. 2007.  No. 970.39 | Spectrophotometric method |
| **Potassium** | 36.5 | 32.5 | mg/100 g | AOAC. 2005.  No. 974.14 | Atomic absorption spectrometry |
| **Selenium** | <100 | <100 | Ppb | Method CFAN/ORS/DBC/CHCB. FDA 2011 | ICP-MS |
| **Sodium** | 1.9 | 1.85 | mg/100 g | AOAC. 2005.  No. 974.14 | Atomic absorption spectrometry |
| **Zinc** | 450 | 405 | Ppb | Method CFAN/ORS/DBC/CHCB. FDA 2011 | ICP-MS |
| **Organic acid profile:** |  |  |  |  |  |
| **Acetic acid** | <0.001 | <0.001 | % | AOAC. 2000.  No. 986.13 | HPLC |
| **Butyric acid** | <0.001 | <0.001 | % | AOAC. 2000.  No. 986.13 | HPLC |
| **Citric acid** | 0.0065 | 0.0060 | % | AOAC. 2000.  No. 986.13 | HPLC |
| **Fumaric acid** | 0.002 | 0.002 | % | AOAC. 2000.  No. 986.13 | HPLC |
| **Gluconic acid** | <0.001 | <0.001 | % | AOAC. 2000.  No. 986.13 | HPLC |
| **Lactic acid** | <0.001 | <0.001 | % | AOAC. 2000.  No. 986.13 | HPLC |
| **Malic acid** | 0.0175 | 0.0150 | % | AOAC. 2000.  No. 986.13 | HPLC |
| **Oxalic acid** | 0.003 | 0.003 | % | AOAC. 2000.  No. 986.13 | HPLC |
| **Propionic acid** | <0.001 | <0.001 | % | AOAC. 2000.  No. 986.13 | HPLC |
| **Quinic acid** | <0.001 | <0.001 | % | AOAC. 2000.  No. 986.13 | HPLC |
| **Succinic acid** | <0.001 | <0.001 | % | AOAC. 2000.  No. 986.13 | HPLC |
| **Tartaric acid** | <0.001 | <0.001 | % | AOAC. 2000.  No. 986.13 | HPLC |
|  |  |  |  |  |  |
| **Crude fiber** | 0 | 0 | % | AOAC. 1990.  No. 962.09 | Gravimetric analysis |
| **Fructose** | 0 | 0 | % | AOAC. 1995.  No. 996.04 | Ion chromatography |
| **Lactose** | 0 | 0 | % | AOAC. 1995.  No. 996.04 | Ion chromatography |
| **Maltose** | 0 | 0 | % | AOAC. 1995.  No. 996.04 | Ion chromatography |
| **Sucrose** | 0 | 0 | % | AOAC. 1995.  No. 996.04 | Ion chromatography |
| **Vitamin A** | 0 | 0 | IU/100 g | AOAC. 2001.  No. 292.13 | HPLC |
| **Vitamin B9**  **(folic acid)** | 0 | 0 | µg/100 g | R-Biopharm. 2011. VitaFast Folic Acid | Microbiological assay |
| **Vitamin C** | 0 | 0 | mg/100 g | Brause *et al*. 2003 | HPLC |
| **Vitamin E** | 0 | 0 | mg/100 g | AOAC. 2000.No. 992.03 | HPLC |
| **Retinol** | 0 | 0 | IU/100 g | AOAC. 2002.  No. 938.04/2001.13 | HPLC |
| **Total omega-3** | n/a | n/a | mg/100 g sample | AOCS. 2002.  No. Ce 1f-96 | Ion chromatography |
| **Total monounsaturated fat** | n/a | n/a | g/100 g sample | AOCS. 2002.  No. Ce 1f-96 | Ion chromatography |
| **Total polyunsaturated fat** | n/a | n/a | g/100 g sample | AOCS. 2002.  No. Ce 1f-96 | Ion chromatography |
| **Total saturated fat** | n/a | n/a | g/100 g sample | AOCS. 2002.  No. Ce 1f-96 | Ion chromatography |
| **Total trans fat** | n/a | n/a | g/100 g sample | AOCS. 2002.  No. Ce 1f-96 | Ion chromatography |
| **Alpha linolenic (ALA)** | n/a | n/a | mg/100 g sample | AOCS. 2002.  No. Ce 1f-96 | Ion chromatography |
| **Docosahexaenoic (DHA)** | n/a | n/a | mg/100 g sample | AOCS. 2002.  No. Ce 1f-96 | Ion chromatography |
| **Docosapentaenoic** | n/a | n/a | mg/100 g sample | AOCS. 2002.  No. Ce 1f-96 | Ion chromatography |
| **Docosatrienoic** | n/a | n/a | mg/100 g sample | AOCS. 2002.  No. Ce 1f-96 | Ion chromatography |
| **Eicosapentaenoic (w-3) (EPA)** | n/a | n/a | mg/100 g sample | AOCS. 2002.  No. Ce 1f-96 | Ion chromatography |
| **Eicosatrienoic (11,14,17)** | n/a | n/a | mg/100 g sample | AOCS. 2002.  No. Ce 1f-96 | Ion chromatography |
|  |  |  |  |  |  |
| AOAC= Official methods of analysis of the Association of Official Analytical Chemists, U.S.A. | | | | | |
| AOCS= Official methods of analysis of the American Oil Chemists’ Society, U.S.A.  HPLC= High-performance liquid chromatography.  ICP-MS= Inductively coupled plasma-mass spectrometry. | | | | | |
| ^a^ Calculated data based on specific standards.  n/a= Not available. | | | | | |
|  | | | | | |
