# Supplementary material for: Hypocholesterolemic Properties and Prebiotic Effects of Mexican Ganoderma lucidum in C57BL/6 Mice
Source: PLoS One. 2016 Jul 20;11(7):e0159631. doi: 10.1371/journal.pone.0159631 (PMC4954724; doi:10.1371/journal.pone.0159631)
Supplement: S2 Table — (DOCX) [file pone.0159631.s003.docx]

| **Gene** | **Sequence** | **Temperature**  **(^o^C)** |
| --- | --- | --- |
| β2M | Forward 5´-TTCTGGTGCTTGTCTCACTG | 61.8 |
|  | Reverse 5´-TATGTTCGGCTTCCCATTCT | 63.3 |
| Srebp1c | Forward 5´-AGACAAACTGCCCATCCACC | 66.2 |
|  | Reverse 5´-AAGCGGATGTAGTCGATGGC | 66.3 |
| Srebp2 | Forward 5´-GATGATCACCCCGACGTTCA | 68.3 |
|  | Reverse 5´-GTCACGAGGCTTTGCACTTG | 66.0 |
| Hmgcr | Forward 5´-GGGTATTGCTGGCCTCTTCA | 66.2 |
|  | Reverse 5´-GGATTGCCATTCCACGAGCT | 68.5 |
| Fasn | Forward 5´-CAAAGGACCAAGCATTGCCC | 68.7 |
|  | Reverse 5´-TACAACAGCCTCAGAGCGAC | 63.2 |
| Acaca | Forward 5´-GCCTCTTCCTGACAAACGAG | 63.9 |
|  | Reverse 5´-GACTGTGCCTGGAACCTCTT | 63.3 |
| Abcg5 | Forward 5´-GATGAGCCAACCACAGGACT | 64.2 |
|  | Reverse 5´-GAAGCCAAGCATCTCCTCTG | 64.0 |
| Abcg8 | Forward 5´-CCTCTCAGGTGCCTTGGTTT | 65.4 |
|  | Reverse 5´-ACGTCGAGTAGTGAGGCTCT | 60.7 |
| Ldlr | Forward 5´-ATGAGTCCCCAGAGACATGC | 64.2 |
|  | Reverse 5´-GAGCCATCTAGGCAATCTCG | 63.8 |
| Abca1 | Forward 5´-AGCCAGAAGGGAGTGTCAGA | 61.1 |
|  | Reverse 5´-GGGAAACAGCCCAGTCAGTA | 64.0 |
| Cyp7a1 | Forward 5´-TGGGCTGTGCTCTAAGTTC | 65.6 |
|  | Reverse 5´-CTGTGTCCAAATGCCTTCGC | 67.9 |

**Supplementary Table 2. Primer sequences used for reverse transcription polymerase chain reaction (RT-PCR).**

β2M: Beta-2 microglubulin. Srebp1c: Sterol regulatory element-binding protein 1. Srebp2: Sterol regulatory element-binding protein 2. Hmgcr: 3-hydroxy-3-methyl-glutaryl-CoA reductase. Fasn: Fatty acid synthase. Acaca: Acetyl-CoA carboxylase. Abcg5: ATP-binding cassette, sub-family G, member 5. Abcg8: ATP-binding cassette, sub-family G, member 8. Ldlr: Low-density lipoprotein receptor. Abca1: ATP-binding cassette, sub-family A. Cyp7a1: Cytochrome P450, family 7.
