# Supplementary material for: Hypocholesterolemic Properties and Prebiotic Effects of Mexican Ganoderma lucidum in C57BL/6 Mice
Source: PLoS One. 2016 Jul 20;11(7):e0159631. doi: 10.1371/journal.pone.0159631 (PMC4954724; doi:10.1371/journal.pone.0159631)
Supplement: S3 Table — (DOCX) [file pone.0159631.s004.docx]

| **Level** | **Sequence** | **Temperature**  **(^o^C)** | |
| --- | --- | --- | --- |
| ***Lactobacillus*** | F: 5'-AGCAGTAGGGAATCTTCCA | | 58.8 |
|  | R: 5'-CACCGCTACACATGGAG | | 62.8 |
| **Universal gene** | F: 5´-AAACTCAAAKGAATTGACGG | | 61.2 |
|  | R: 5'-CTCACRRCACGAGCTGAC | | 56.9 |

**Supplementary Table 3. The 16S rRNA gene-targeted primers used for microbiota analysis in the genus *Lactobacillus.***

F= Forward. R= Reverse.
